# CCR9+ and CD103+ tolerogenic dendritic cell populations in food allergy patients undergoing oral immunotherapy

**DOI:** 10.1186/2045-7022-1-S1-O51

**Published:** 2011-08-12

**Authors:** Marco Garcia, Amit Singh, Grace Yu, Robi Bucayu, Trevor Longbottom, Kari Nadeau

**Affiliations:** 1 Stanford University, Biology, Stanford, USA; 2 Stanford University, Allergy and Immunology, Stanford, USA; 3 Stanford University, Pediatrics, Stanford, USA

## Rationale

CD103+ DCs (dendritic cells) and CCR9+ pDCs (plasmacytoid DCs) have been implicated in promoting tolerance to antigens through regulatory-T cell induction. We have conducted food oral immunotherapy (OIT) clinical studies for the last 3 years at Stanford University. We hypothesized that subjects with food allergies have low CD103+ and CCR9+ expression on their DCs but that these DC populations change over time while on therapy.

## Methods

OIT was conducted and blood samples were drawn at baseline and approximately every 5 months during the study. The study is currently ongoing. PBMCs (peripheral blood mononuclear cells) were purified and flow cytometry was performed on gated DCs (LSRII, BD Biosciences).

## Results

DCs expressing CD103 (integrin-alpha E) and CCR9 (CCL25 chemokine receptor) were examined in three cohorts - (1) patients undergoing milk or peanut OIT (n=8), (2) healthy controls (HC) (n=8), and (3) non-OIT food allergy patients (FA) (n=8). PBMCs were incubated for 6 or 18 hours either with or without offending allergen. CCR9+ expression on pDCs was significantly greater in HC versus FA patients (42%±25% vs 11%±10%; p <0.01) while CD103 expression on DCs was comparatively greater in HC versus FA patients (0.19%±0.17% vs 0.07%±0.07%; p=0.16). After offending allergen stimulation for both 6 and 18 hours, CCR9 presence on pDCs significantly increased more in FA patients than in HC patients (213MFI*-6hr, 188MFI-18hr versus 12MFI for HC; p<0.03). In OIT patients, CCR9 change on pDCS after stimulation was significantly different than their baseline CCR9 MFI shift values (16MFI versus 188MFI; p<0.03) and more in line with the HC profile. *-Median Fluorescent Intensity.

## Conclusion

The CCR9 and CD103 DC populations may play an important role for food allergy patients undergoing OIT. These tolerogenic DC changes in OIT may reveal one way that regulatory T-cell mediated tolerance, T-cell anergy, and/ or clonal deletion is induced.

